# Protective effect of glycyrrhizin, a direct HMGB1 inhibitor, on post-contrast acute kidney injury

**DOI:** 10.1038/s41598-021-94928-5

**Published:** 2021-08-02

**Authors:** Hyewon Oh, Arom Choi, Nieun Seo, Joon Seok Lim, Je Sung You, Yong Eun Chung

**Affiliations:** 1 Department of Radiology, Severance Hospital, Yonsei University College of Medicine, 50-1 Yonsei-ro, Seodaemun-gu, Seoul, 03722 Republic of Korea; 2 Department of Emergency Medicine, Yonsei University College of Medicine, 211 Eonju-Ro, Gangnam-Gu, Seoul, 06273 Republic of Korea

**Keywords:** Immunology, Molecular medicine, Nephrology

## Abstract

Post contrast-acute kidney injury (PC-AKI) is defined as the deterioration of renal function after administration of iodinated contrast media. HMGB1 is known to play an important role in the development of acute kidney injury. The purpose of this study was to investigate the association between HMGB1 and PC-AKI and the protective effect of glycyrrhizin, a direct inhibitor of HMGB1, in rats. Rats were divided into three groups: control, PC-AKI and PC-AKI with glycyrrhizin. Oxidative stress was measured with MDA levels and H_2_DCFDA fluorescence intensity. The mRNA expressions of pro-inflammatory cytokines (IL-1α, IL-1β, IL-6 and TNF-α) and kidney injury markers (KIM-1, NGAL and IL-18) were assessed using RT-PCR and ELISA in kidney tissue. In addition, the serum and intracellular protein levels of HMGB1were analyzed with the enzyme-linked immunosorbent assay (ELISA) and western blotting. Histologic changes were assessed with H&E staining using the transmission electron microscope (TEM). Moreover, serum creatinine (SCr), blood urea nitrogen (BUN) and lactate dehydrogenase (LDH) levels were assessed. Oxidative stress, pro-inflammatory cytokines, kidney injury markers and LDH were significantly higher in PC-AKI compared to the controls, but were lower in PC-AKI with glycyrrhizin. Intracellular and serum HMGB1 levels significantly increased after contrast media exposure, whereas they markedly decreased after glycyrrhizin pretreatment. SCr and BUN also decreased in PC-AKI with glycyrrhizin compared to PC-AKI. In PC-AKI, we could frequently observe tubular dilatation with H&E staining and cytoplasmic vacuoles on TEM, whereas these findings were attenuated in PC-AKI with glycyrrhizin. Our findings indicate that HMGB1 plays an important role in the development of PC-AKI and that glycyrrhizin has a protective effect against renal injury and dysfunction by inhibiting HMGB1 and reducing oxidative stress.

## Introduction

Post-contrast acute kidney injury (PC-AKI) occurs in approximately 5–6% of patients after administration of intravenous contrast media and this incidence steeply increases in patients with impaired renal function^1^. PC-AKI is the third most common cause of acute renal failure in hospitalized patients^2,3^ and can cause long-term adverse effects including loss of kidney function and increased morbidity and mortality^4^. Although the pathogenesis of PC-AKI is not yet fully elucidated, suggested causes of PC-AKI are the direct toxicity of contrast media, contrast media-driven reactive oxygen species and/or renal hypoxia due to vasoconstriction or high viscosity of the contrast media itself^2,3^.

The high-mobility group box 1 (HMGB1) exists in the nuclei of mammalian cells and is a novel member of the damage-associated molecular pattern (DAMP) family. HMGB1 can be actively released from the nucleus into the cytoplasm and extracellular space in response to sterile inflammation and infection when cell membrane receptors interact with microbe-associated molecular patterns, pathogen-associated molecular patterns and inflammatory mediators such as tumor necrosis factor (TNF-α), IL-1 and interferon-γ. HMGB1 can also be passively released from necrotic or apoptotic cells^5,6^. After excretion from the cell, HMGB1 acts as a pro-inflammatory mediator when it binds to cell surface receptors including advanced glycation end products (RAGE) or toll-like receptors (TLR) and stimulates immunocompetent cells to produce pro-inflammatory cytokines such as TNF-α, IL-1, IL-6 and IL-8, resulting in symptoms such as fever, anorexia and other inflammatory responses^5,6^. HMGB1 also plays an important role in the pathogenesis of various diseases including sepsis, gastrointestinal inflammation, respiratory disorders, autoimmune diseases, hemorrhagic shock, cerebral ischemia and myocardial infarction^6^. HMGB1 is also involved in renal disease including acute kidney injury because HMGB1 can promote kidney injury through the TLR4 pathway^7,8^.

Glycyrrhizin is derived from licorice root extract and binds directly to the high-mobility group 1 (HMG1) box in HMGB1, consequently inhibiting the chemoattractant and mitogenic activity of HMGB1^9–11^. In AKI caused by ischemia–reperfusion injury, glycyrrhizin is thought to attenuate renal injury through this inhibition^12^. As PC-AKI is classified as acute kidney injury, HMGB1 might be a factor in the development of PC-AKI. However, to our knowledge, no studies have explored the role of HMGB1 and the protective effect of glycyrrhizin against PC-AKI. So, the purpose of this study was to investigate the association between HMGB1 and PC-AKI and the protective effect of glycyrrhizin.

## Results

### Glycyrrhizin-mitigated oxidative stress in PC-AKI

**Figure 1:**
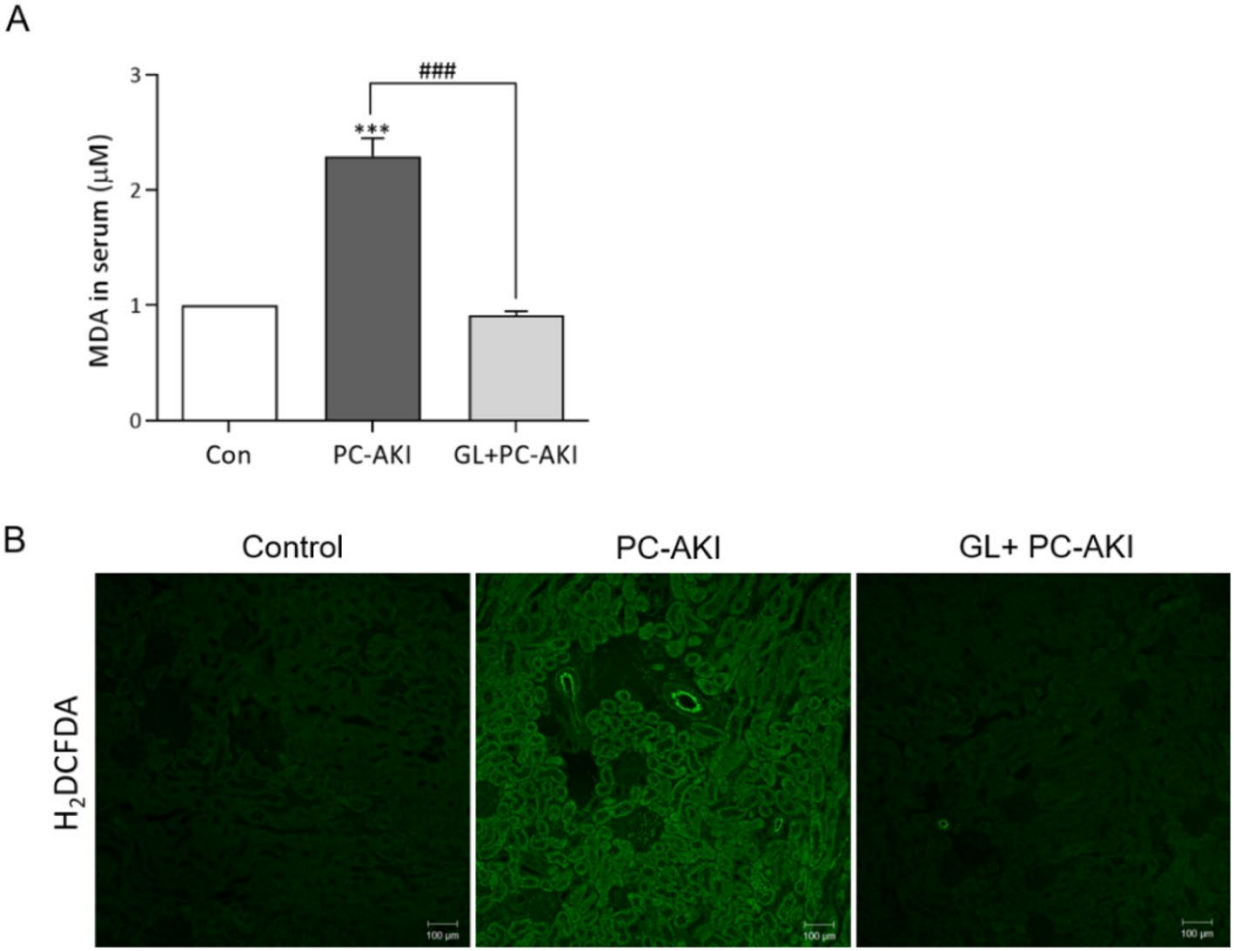
Glycyrrhizin attenuates oxidative stress. Oxidative stress was evaluated with MDA levels in the serum (**A**) and H_2_DCFDA staining in kidney tissue (**B**). Oxidative stress was significantly higher in the PC-AKI group compared to the controls, whereas it was mitigated in the PC-AKI with glycyrrhizin group. Magnification = 10X, Scale bar = 100 μm. Results were expressed as means ± SEMs. N = 8 for each group. Statistical significance: ***P < 0.001 Con vs PC-AKI, ^###^*P* < 0.001 PC-AKI vs GL + PC-AKI. Abbreviation: PC-AKI, post-contrast acute kidney injury; GL, glycyrrhizin; MDA, malondialdehyde.

We confirmed the effect of glycyrrhizin on reactive oxygen species (ROS) in the PC-AKI group. The MDA levels were higher in the PC-AKI group compared to the controls (*P* < 0.001), whereas MDA levels were lower in the PC-AKI with glycyrrhizin group compared to the PC-AKI group (*P* < 0.001) (Fig. 1A). In addition, in H_2_DCFDA staining, the fluorescence intensity of the PC-AKI group was markedly higher than the controls, whereas it was lower after glycyrrhizin pretreatment (Fig. 1B).

### Effect of glycyrrhizin on the protein expression of HMGB1 in PC-AKI

**Figure 2:**
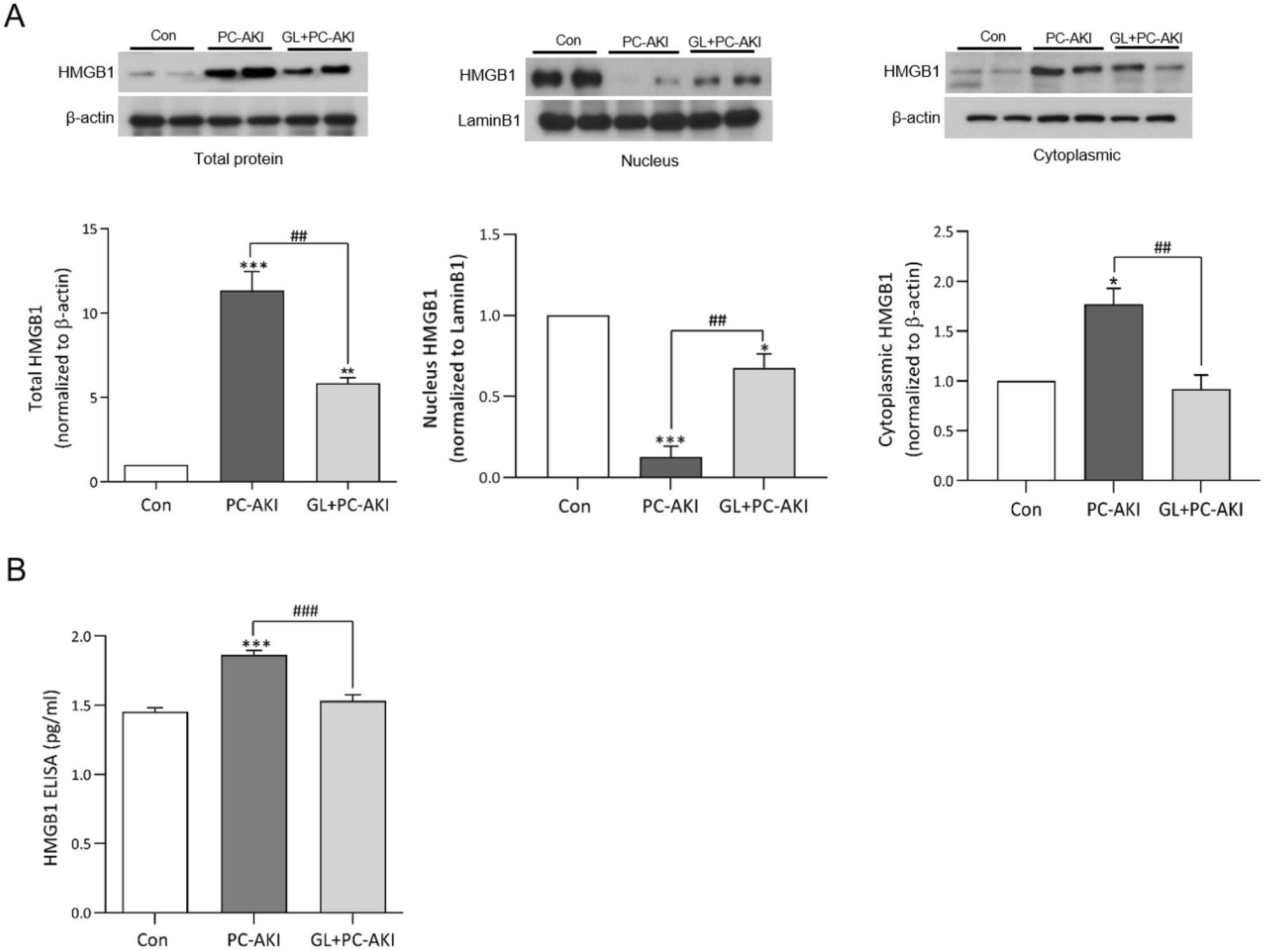
Effect of glycyrrhizin on HMGB1 protein expression. (A) Representative western blot bands of HMGB1 with intracellular total protein, nucleus and cytoplasmic protein isolation. Expression levels were normalized to β-actin. The expressions of intracellular and cytoplasmic HMGB1 were significantly increased in the PC-AKI group compared to the controls, and were significantly reduced in the PC-AKI with glycyrrhizin group. On the other hand, the expression of nucleus HMGB1 in PC-AKI group significantly reduced compared to the control, but was significantly increased PC-AKI with glycyrrhizin group. The β-actin was used as a cytoplasmic and intracellular marker and LaminB1 as a nuclear marker. (B) The serum HMGB1 measured by ELISA was significantly higher in the PC-AKI group compared to the controls, but was significantly lower in the PC-AKI with glycyrrhizin group. Images of blots were cropped. Full-length blots are presented in Supplementary Figure 1. Results were expressed as means ± SEMs. N = 8 for each group. Statistical significance: *P < 0.05, ***P < 0.001 Con vs PC-AKI and Con vs PC-AKI + GL, ^##^*P* < 0.01 and ^###^*P* < 0.001 PC-AKI vs GL + PC-AKI. Abbreviation: PC-AKI, post-contrast acute kidney injury; GL, glycyrrhizin.

We evaluated whether glycyrrhizin could inhibit the protein expression of HMGB1. The protein expression of intracellular (*P* < 0.001), cytoplasmic (*P* = 0.010) HMGB1 increased in the PC-AKI group compared to the controls, whereas the PC-AKI with glycyrrhizin group showed lower protein expression levels than the PC-AKI group (intracellular; *P* = 0.004; cytoplasmic; *P* = 0.008). Conversely, the expression of nuclear HMGB1 decreased in the PC-AKI group compared to the controls (nucleus; *P* < 0.001), whereas it significantly increased in the PC-AKI with glycyrrhizin group compared to the PC-AKI group (nucleus; *P* = 0.003). (Fig. 2A). Full-length blots are displayed in Supplementary Figure 1. Serum HMGB1 concentrations were also higher in the PC-AKI group compared to the controls (*P* < 0.001). Serum HMGB1 concentrations were lower in the PC-AKI with glycyrrhizin group than the PC-AKI group (*P* < 0.001) and similar to that of the controls (Fig. 2B).

### Effect of glycyrrhizin on the mRNA expression of pro-inflammatory cytokines in PC-AKI

**Figure 3:**
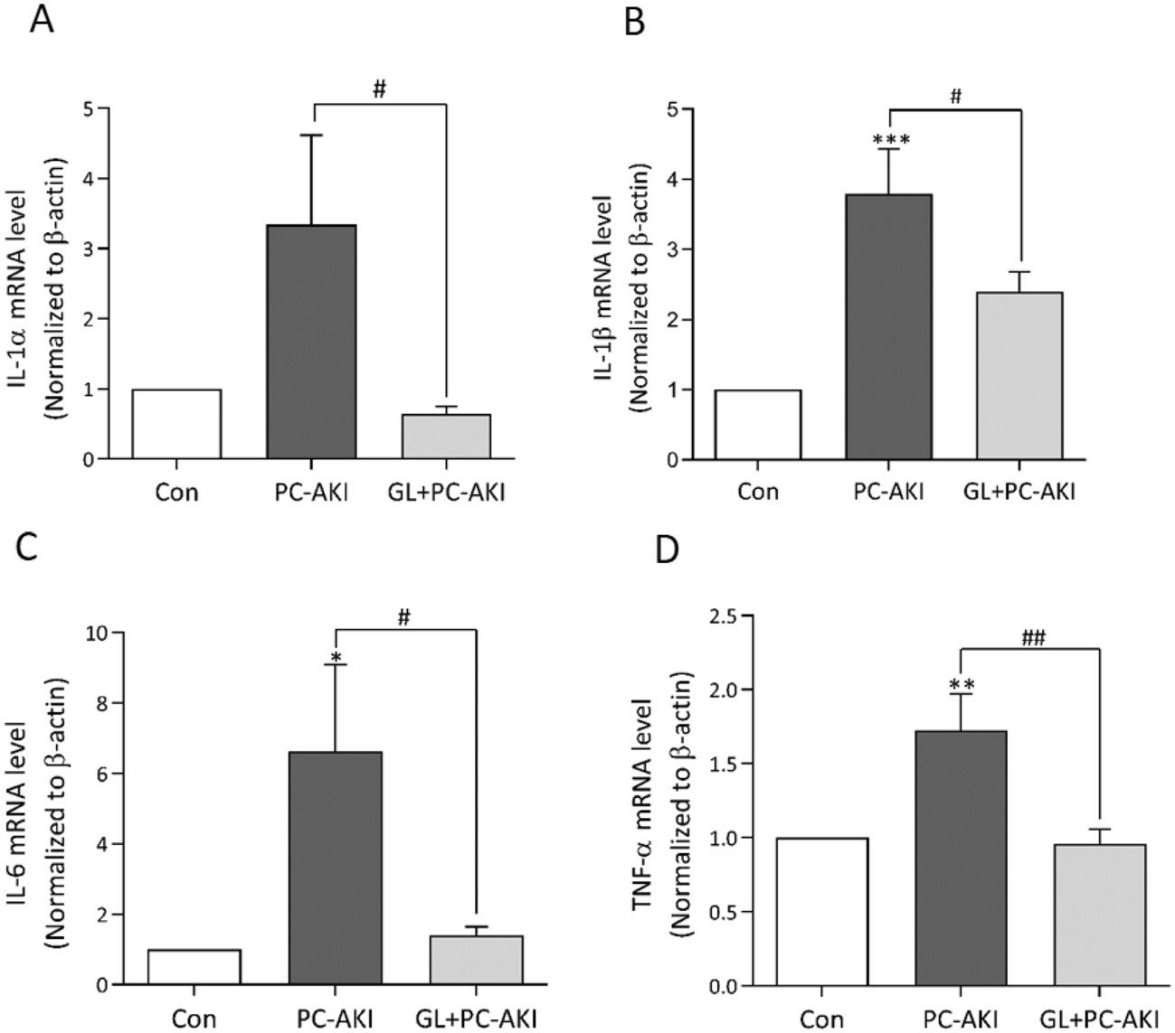
Effect of glycyrrhizin on the mRNA expression of pro-inflammatory cytokines. (A-D) Relative expressions of IL-1α, IL-1β, IL-6 and TNF-α mRNA were analyzed using RT-PCR. All pro-inflammatory cytokines were higher in PC-AKI compared to the controls with/without statistical significance, whereas they were lower in the glycyrrhizin pretreatment group. Results were expressed as means ± SEMs. N = 8 for each group. Statistical significance: *P < 0.05, **P < 0.01 and ***P < 0.001 Con vs PC-AKI. ^#^*P* < 0.05 and ^##^*P* < 0.01 PC-AKI vs GL + PC-AKI. Abbreviation: PC-AKI, post-contrast acute kidney injury; GL, glycyrrhizin.

To examine the protective effect of glycyrrhizin on the inflammatory process, we assessed mRNA expression levels using RT-PCR. The mRNA expressions of IL-1α, IL-1β, IL-6 and TNF-α were all significantly upregulated in the PC-AKI group compared to the controls (IL-1α; *P* = 0.09, IL-1β; *P* < 0.001, IL-6; *P* = 0.020 and TNF-α; *P* = 0.004). As expected, the expression of all pro-inflammatory cytokines decreased in the PC-AKI with glycyrrhizin group compared to the PC-AKI group. (IL-1α; *P* = 0.040, IL-1β; *P* = 0.040, IL-6; *P* = 0.040 and TNF-α; *P* = 0.003) (Fig. 3A–D).

### Glycyrrhizin-mitigated kidney injury in PC-AKI

**Figure 4:**
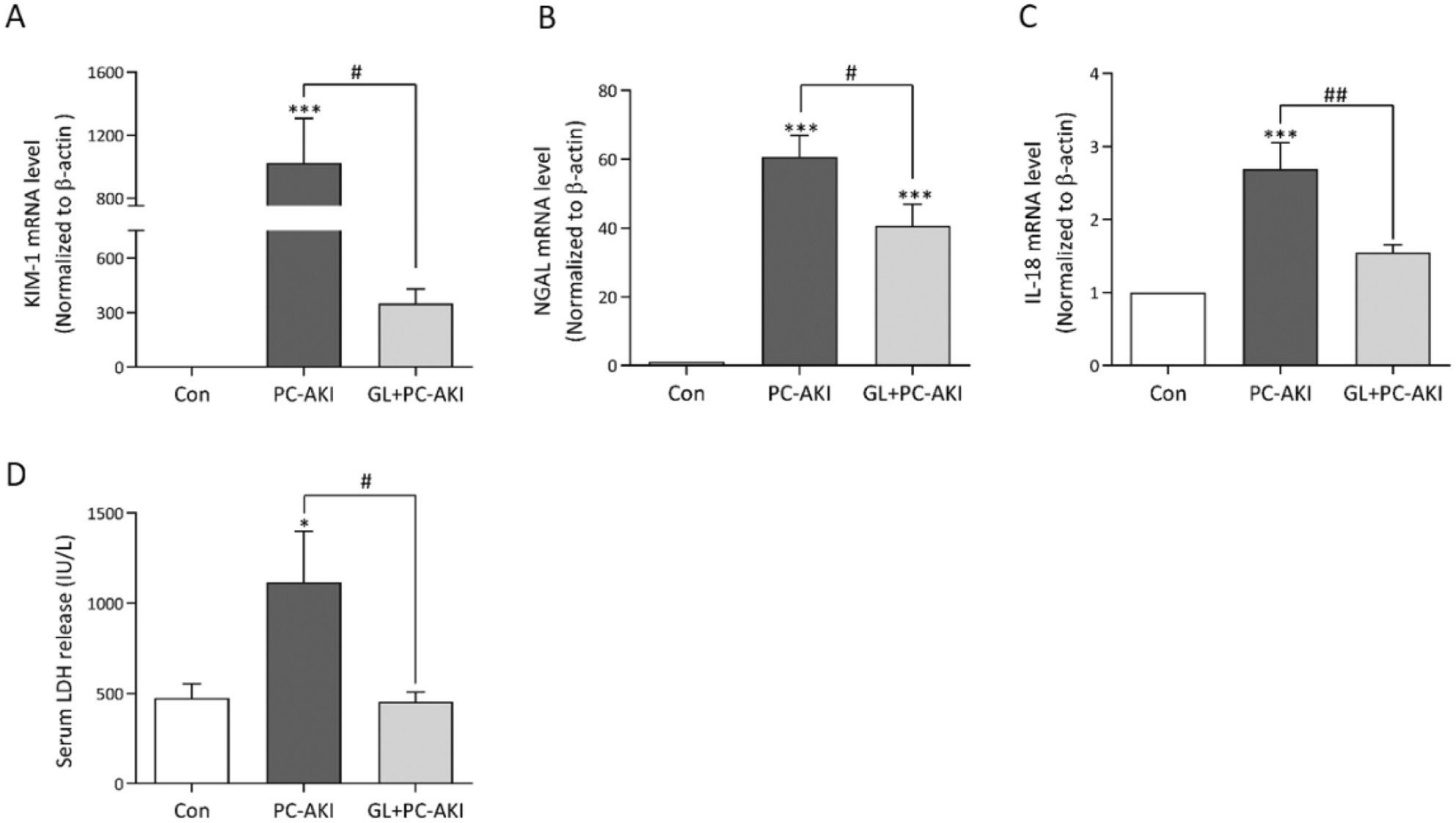
Effect of glycyrrhizin on the mRNA expression of kidney injury markers and serum lactate dehydrogenase (LDH). (A-C) Relative expressions of KIM-1, NGAL and IL-18 mRNA were measured using RT-PCR. The mRNA expression of kidney injury markers was significantly higher in the PC-AKI group compared to the controls, whereas it was significantly lower in the PC-AKI with glycyrrhizin group. (D) Compared to the control group, serum LDH levels significantly increased in PC-AKI, whereas they decreased with glycyrrhizin pretreatment. Results were expressed as means ± SEMs. N = 8 for each group. Statistical significance: *P < 0.05 and ***P < 0.001 Con vs PC-AKI and Con vs PC-AKI + GL, ^#^*P* < 0.05 and ^##^*P* < 0.01 PC-AKI vs GL + PC-AKI. Abbreviation: PC-AKI, post-contrast acute kidney injury; GL, glycyrrhizin; LDH, lactate dehydrogenase.

Next, we evaluated the mRNA expression levels of kidney injury markers using RT-PCR. The expressions of KIM-1, NGAL and IL-18 were higher in the PC-AKI group compared to the controls (*P* < 0.001). Pretreatment with glycyrrhizin seemed to reduce the expression levels of these genes compared to the PC-AKI group (KIM-1 and NGAL; *P* = 0.020, IL-18; *P* = 0.001) (Fig. 4A–C). In addition, we further investigated whether NSAID and L-NAME could affect the kidney and found that there were no significant differences in renal function (Scr and BUN) and kidney injury markers between the controls and the NSAID + L-NAME group (*P* > 0.999) (Supplementary Figure 2). We also confirmed urinary KIM-1 and serum IL-18 levels using ELISA. We found that the concentrations of KIM-1 and IL-18 significantly higher in the PC-AKI group compared to the controls (*P* < 0.001), whereas they were lower in the PC-AKI with glycyrrhizin group compared to the PC-AKI group (KIM-1; *P* < 0.001 and IL-18; *P* = 0.008). (Supplementary Figure 3). In addition, serum LDH was assessed as an early renal damage biomarker. LDH concentrations were higher in the PC-AKI group compared to the controls (*P* < 0.05). On the other hand, LDH levels were lower in PC-AKI pretreated with glycyrrhizin (*P* = 0.040) (Fig. 4D).

**Figure 5:**
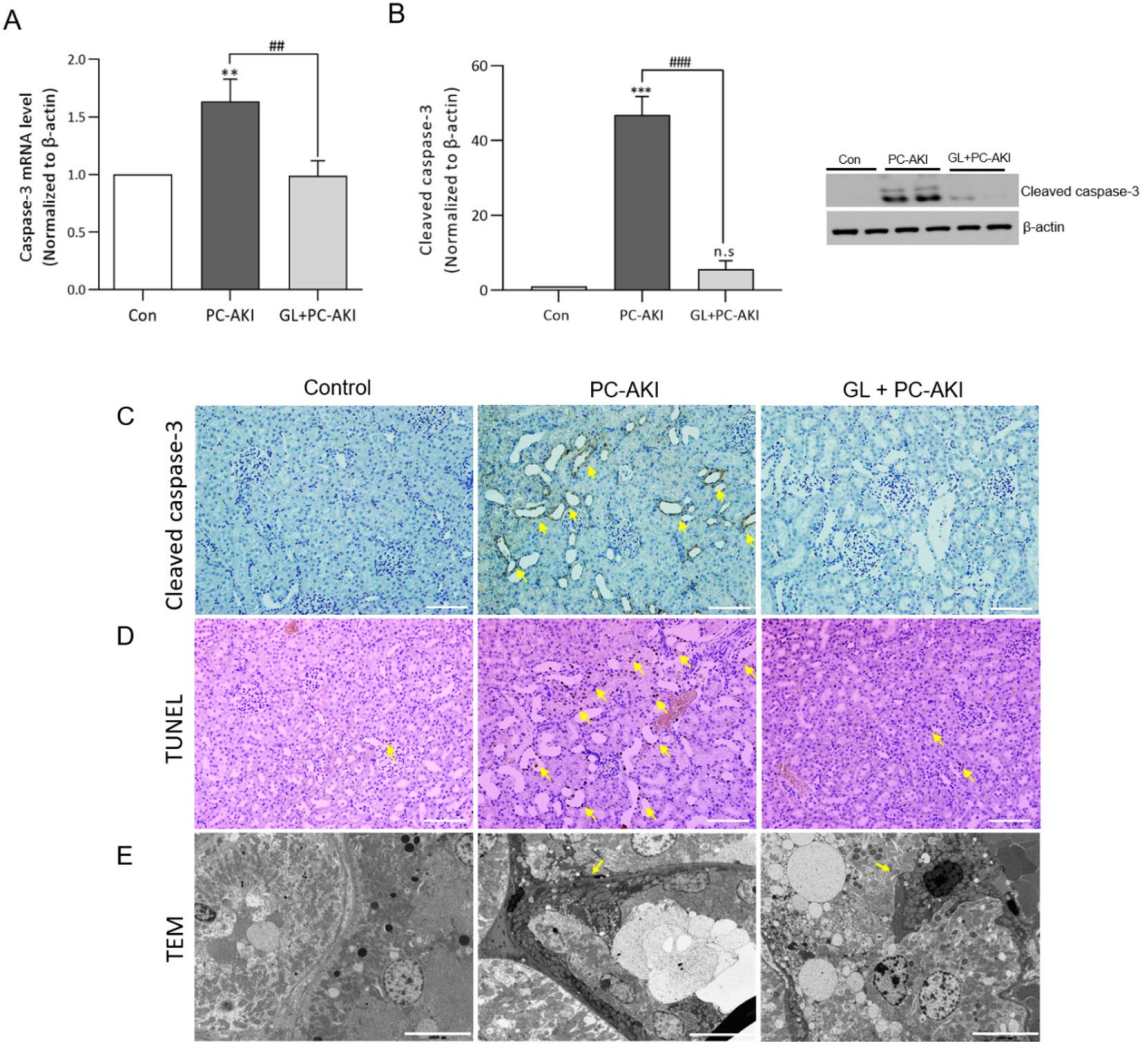
Glycyrrhizin-mitigated apoptosis by PC-AKI. Relative expressions of caspase-3 mRNA (A) and protein levels of cleaved caspase-3 (B) were significantly increased in the PC-AKI group compared to the controls, whereas they were significantly lower in the PC-AKI with glycyrrhizin group than the PC-AKI group. (C-D) The immunohistochemistry of cleaved caspase-3 and TUNEL indicated apoptotic cells (arrowheads) in the PC-AKI group. Magnification = 20X, Scale bar = 100 μm. (E) TEM images shows that the area of apoptosis (arrows) in the kidney tissue of PC-AKI is wider than that of the PC-AKI with glycyrrhizin group. Magnification = 3 K, Scale bar = 5000 nm. Images of blots were cropped. Full-length blots are presented in Supplementary Figure 1. Statistical significance: **P < 0.01 and ***P < 0.001 Con vs PC-AKI, ^##^*P* < 0.01 and ^###^*P* < 0.001 PC-AKI vs PC-AKI + GL. Abbreviation: PC-AKI, post-contrast acute kidney injury; GL, glycyrrhizin; TEM, transmission electron microscopy; TUNEL, Terminal deoxynucleotidyl transferase (TdT)-mediated dUTP nick-end-labeling.

We evaluated the degree of apoptosis by the caspase-3 mRNA and cleaved caspase-3 protein levels. Apoptosis was significantly increased in the PC-AKI group compared to the controls, but with significant mitigation in the PC-AKI with glycyrrhizin group compared to the PC-AKI group (caspase3; *P* = 0.002, cleaved caspase-3; *P* < 0.001) (Fig. 5A,B). Immunohistochemistry results for cleaved caspase-3, TUNEL staining and TEM images also indicated that apoptosis increased in the PC-AKI group compared to the controls and that it decreased after pretreatment with glycyrrhizin (Fig. 5C–E). Full-length blots are displayed in Supplementary Figure 1.

### Effect of glycyrrhizin on renal function in PC-AKI

**Figure 6:**
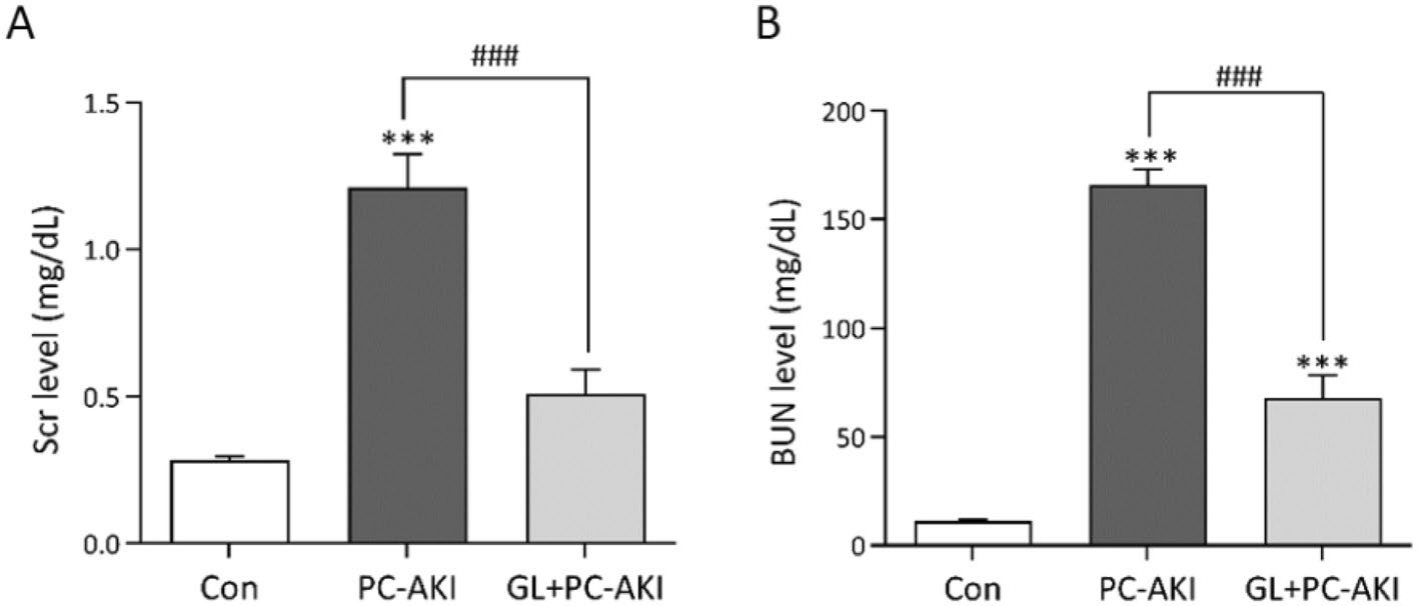
Effect of glycyrrhizin on renal function. (A-B) Compared to the controls, SCr and BUN significantly increased in the PC-AKI group, whereas they decreased in the PC-AKI with glycyrrhizin group compared to the PC-AKI group. Results were expressed as means ± SEMs. N = 8 for each group. Statistical significance: ***P < 0.001, Con vs PC-AKI and Con vs GL + PC-AKI, ^###^*P* < 0.001, PC-AKI vs GL + PC-AKI. Abbreviation: PC-AKI, post-contrast acute kidney injury; GL, glycyrrhizin; Scr, serum creatinine; BUN, blood urea nitrogen.

We also confirmed the protective effect of glycyrrhizin on renal function in PC-AKI. As shown in Fig. 6, SCr and BUN were higher in the PC-AKI group compared to the controls (*P* < 0.001). However, both SCr and BUN were lower in the PC-AKI with glycyrrhizin group than in the PC-AKI group (*P* < 0.001) (Fig. 6A,B).

### Effect of glycyrrhizin on renal histopathology in PC-AKI

**Figure 7:**
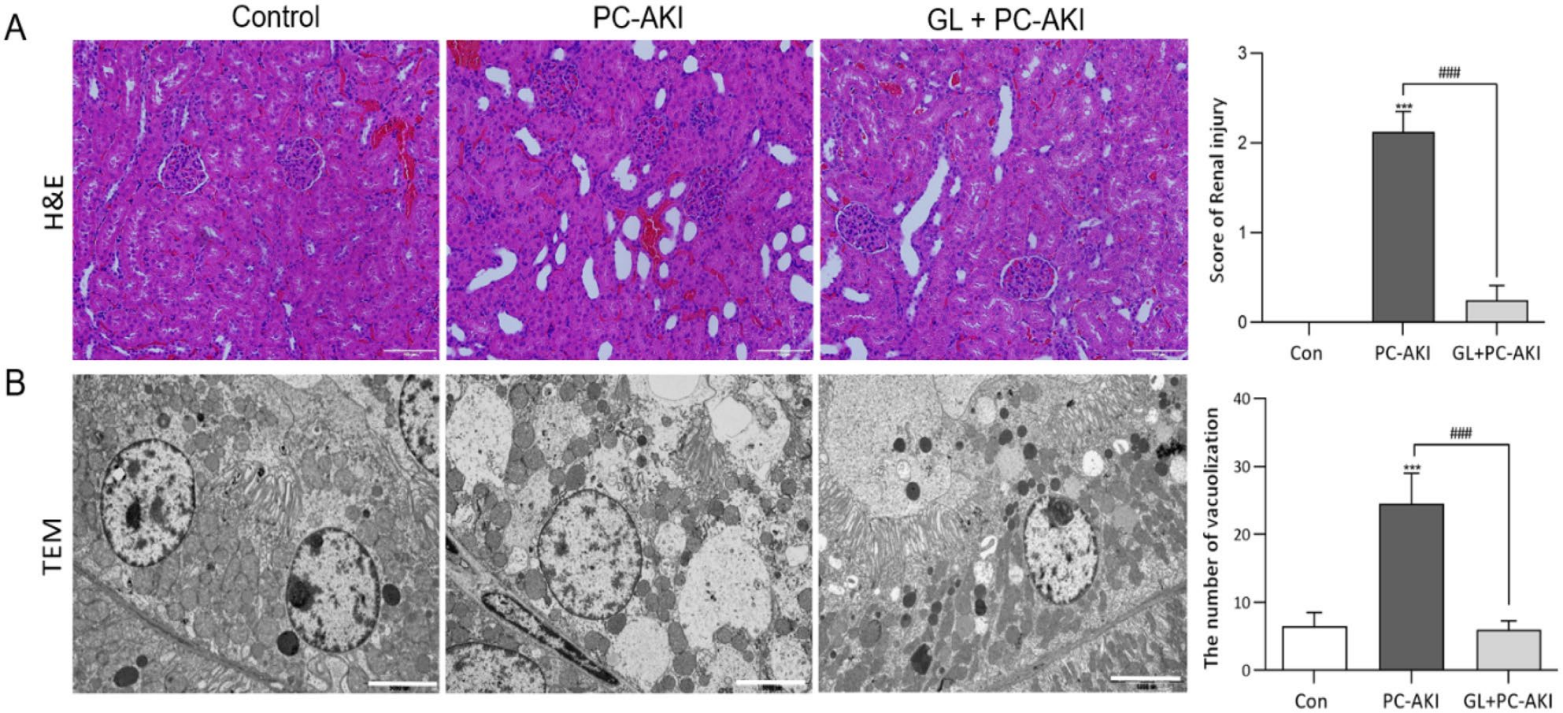
Effect of glycyrrhizin on kidney histology. (A) Histological appearance of kidney tissue after H&E staining. Compared to the controls, renal tubular dilatation was prominent in PC-AKI, whereas it was mitigated in the PC-KAI with glycyrrhizin group. Magnification = 20X, Scale bar = 100 μm. (B) Transmission electron microscopy (TEM) images show multiple large vacuoles in the cytoplasm of the PC-AKI group, but this phenomenon was alleviated in the PC-AKI with glycyrrhizin group. Magnification = 5 K, Scale bar = 5000 nm (B-C). Statistical significance: ***P < 0.001 Con vs PC-AKI, ^###^*P* < 0.001 PC-AKI vs PC-AKI + GL. Abbreviation: PC-AKI, post-contrast acute kidney injury; GL, glycyrrhizin; TEM, transmission electron microscopy.

Histological appearance was compared between the controls, PC-AKI and PC-AKI with glycyrrhizin in Fig. 7. In H&E staining, prominent tubular dilatation was noted in the PC-AKI group. In the PC-AKI with glycyrrhizin group, tubular dilatation was less prominent (Fig. 7A). On TEM, vacuolization was noted in the cytoplasm of tubular cells in PC-AKI and the PC-AKI with glycyrrhizin group had a lower degree of vacuolization than the PC-AKI group (Fig. 7B).

### HMGB1 knock-down inhibiting the expression of HMGB1, pro-inflammatory cytokines, and kidney injury markers after contrast media exposure

**Figure 8:**
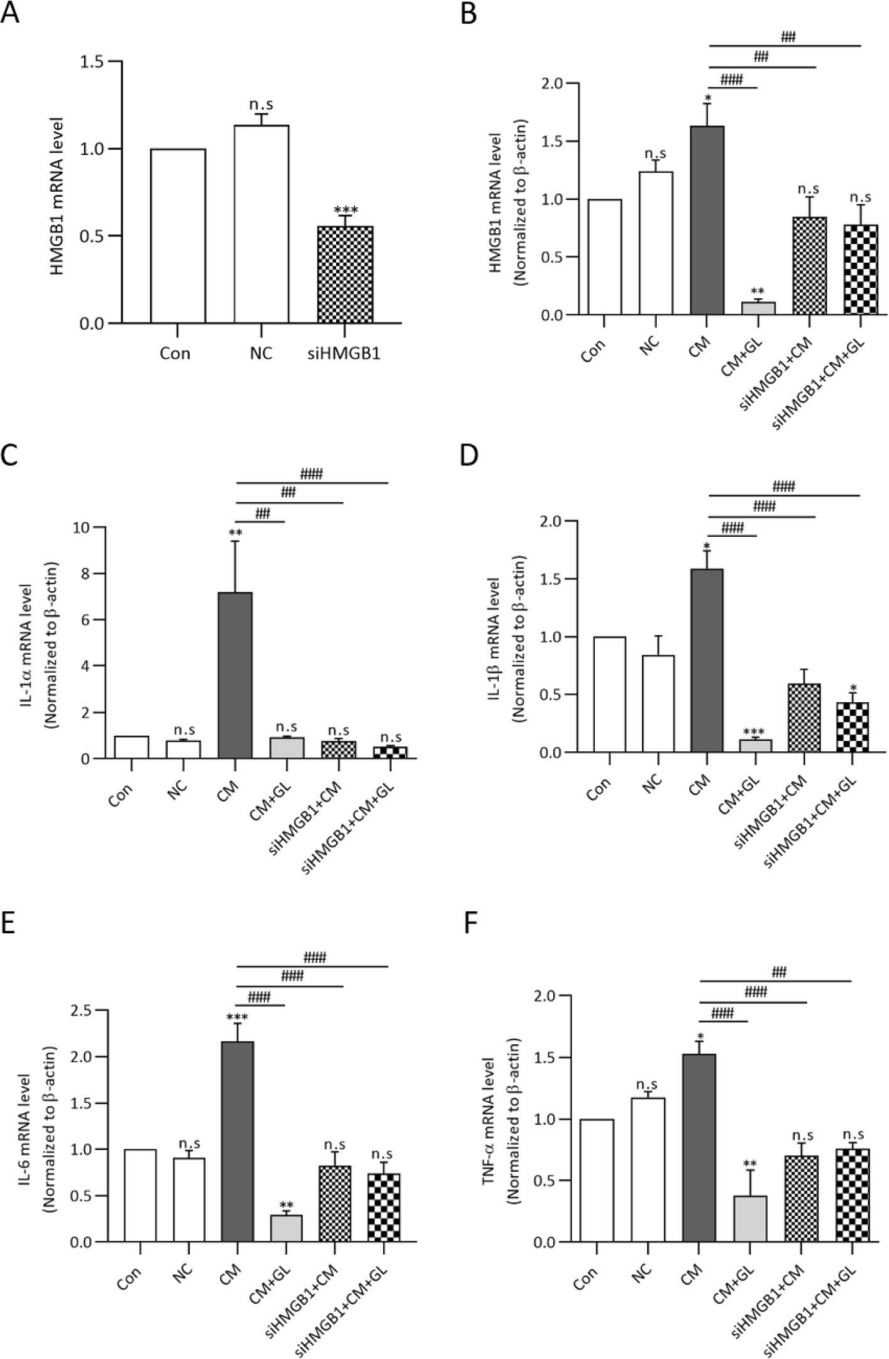
Knocking down HMGB1 inhibits the mRNA expression of HMGB1 and pro-inflammatory cytokines after contrast media exposure. (A) Knocking down HMGB1 inhibits the mRNA expression of HMGB1 compared to the controls and negative control. (B-F) The mRNA expression of HMGB1 and all of the pro-inflammatory cytokines did not significantly increase after cells were exposed to contrast media or contrast media + glycyrrhizin after knocking down HMGB1. (C-F). Results were expressed as means ± SEMs of three independent experiments, which were each performed in duplicate. Statistical significance: *P < 0.05, **P < 0.01 and ***P < 0.001 Con vs CM and Con vs CM + GL, Con vs siHMGB1 ^##^*P* < 0.01 and ^###^*P* < 0.001, CM vs CM + GL, CM vs siHMGB1 + CM, CM vs siHMGB1 + CM + GL. Abbreviation: NC, negative control; CM, contrast media; GL, glycyrrhizin.

**Figure 9:**
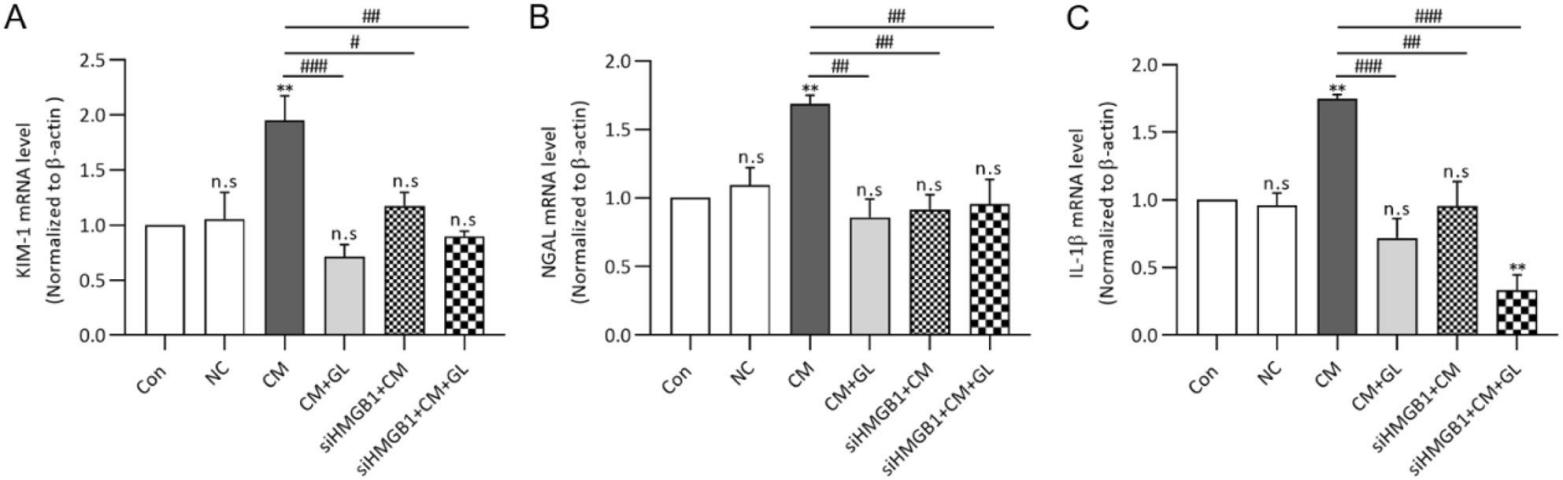
Knocking down HMGB1 inhibits the mRNA expression of kidney injury markers after contrast media exposure. (A-C) The mRNA expressions of KIM-1, NGAL and IL-18 mRNA were measured using RT-PCR. Similar to pro-inflammatory cytokines, the mRNA expression of kidney injury markers did not significantly increase after cells were exposed to contrast media or contrast media + glycyrrhizin after knocking down HMGB1. Results were expressed as means ± SEMs for the three independent experiments, which were each performed in duplicate. Statistical significance: **P < 0.01 Con vs siHMGB1 + CM + GL. ^#^*P* < 0.05, ^##^*P* < 0.01 and ^###^*P* < 0.001, CM vs CM + GL, CM vs siHMGB1 + CM, CM vs siHMGB1 + CM + GL. Abbreviation: NC, negative control; CM, contrast media; GL, glycyrrhizin.

To verify the effect of endogenous HMGB1 on NRK52E cells, the HMGB1 gene was knocked down using siRNA methods (Fig. 8A). The mRNA expressions of HMGB1, all of the pro-inflammatory cytokines and kidney injury markers did not increase after exposure to contrast media with/without glycyrrhizin when HMGB1 was knocked down compared to the controls (Figs. 8B–F and 9).

## Discussion

Our results demonstrated that oxidative stress and inflammatory cytokines including IL-1, IL-6 and TNF-α increased in the PC-AKI model. Kidney injury makers such as KIM-1, NGAL and IL-18 also increased after exposure to contrast media. Furthermore, cytoplasmic and serum HMGB1 increased along with total cellular HMGB1 in the PC-AKI model. In this model, we could see that glycyrrhizin effectively mitigated kidney injury after contrast media exposure. Renal function impairment and histologic change was also attenuated with glycyrrhizin pretreatment. These results suggest that HMGB1 plays an important role in the development of PC-AKI and that glycyrrhizin has a protective effect against PC-AKI.

HMGB1 contributes to the development of many renal diseases including ischemic reperfusion injury, renal fibrosis due to chronic kidney disease, diabetic nephropathy, and granulomatous nephritis^7,8,13^. HMGB1 is also thought to be involved in the development of PC-AKI as increased levels of HMGB1 were found in the supernatants of previous cell experiments after cells were exposed to contrast media^14^. These results were comparable to our animal study which also found increased serum HMGB1 in the PC-AKI group. We also showed that total cellular HMGB1 as well as cytoplasmic and serum HMGB1 increases after the administration of contrast media, suggesting that HMGB1 might be actively translocated from the nucleus to the cytoplasm after contrast media exposure. In previous studies, the extracellular release of HMGB1 upregulated the release of pro-inflammatory cytokines^6,15,16^. This was also noted here as pro-inflammatory cytokines including IL-1α, IL-1β, IL-6 and TNF-α significantly increased in the kidney tissue of the PC-AKI group compared to the controls.

Glycyrrhizin directly binds to HMGB1 by interacting with two concave shallow surfaces and inhibits the chemotactic function of HMGB1^11^. Through this, glycyrrhizin can ameliorate sepsis-induced AKI, renal ischemic-reperfusion injury, gentamicin-induced AKI and diabetic kidney disease^10,12,17–19^. In our results, pro-inflammatory cytokines decreased in the PC-AKI with glycyrrhizin group and SCr and BUN were significantly lower in the PC-AKI with glycyrrhizin group compared to the PC-AKI group. These results suggest that the deterioration of renal function after contrast media exposure could be mitigated with glycyrrhizin by blocking HMGB1 and subsequently, the pro-inflammatory cytokines.

PC-AKI is thought to be caused by both renal hemodynamic changes and direct renal parenchymal damage^20^. The latter might be due to the direct toxicity of iodine contained in the contrast media to the tubular epithelial cells and endothelial cells or due to enhanced production of ROS by the contrast media, resulting in increased oxidative stress^2,20^. Hence, the cytotoxicity of contrast media might be ameliorated by reducing oxidative stress. Glycyrrhizin is one well-known antioxidant^21^. According to previous studies, oxidative stress can be reduced by glycyrrhizin in sepsis- or gentamicin-induced AKI, preventing further renal injury^17,18^. We also demonstrated that glycyrrhizin could reduce oxidative stress after the administration of contrast media and this might partly contribute to the protection of renal function in PC-AKI.

SCr has two innate drawbacks that limit its role in the early diagnosis of AKI. Changes in SCr become apparent slower than benign acute kidney injury due to its large volume of distribution and SCR has a narrow time window for the detection of kidney injury due to its sharp decrease after GFR recovery. Thus, other biomarkers such as NGAL, KIM-1, IL-18 and LDH have been suggested instead of SCr^22,23^. In a previous study, these early biomarkers of AKI also increased in PC-AKI^23–25^. In our study, not only SCr and BUN, but also early biomarkers including the mRNA expression levels of KIM-1, NGAL, IL-18 and serum levels of LDH increased significantly in the PC-AKI group, while significantly decreasing after glycyrrhizin pretreatment. Furthermore, apoptosis was also attenuated by pretreatment with glycyrrhizin. These results suggested that the administration of glycyrrhizin could mitigate kidney injury caused by contrast media.

The vacuolization of tubular epithelial cells is one of the histopathological change of PC-AKI. It is known as an early sign of PC-AKI, although the degree of vacuolization does not correlate with the deterioration of renal function^20,26^. In our study, we observed that prominent vacuolization of tubular cells that was noted in the PC-AKI model was mitigated after glycyrrhizin pretreatment.

Although experimental and clinical studies have found biological and functional evidence of contrast media playing a role in the development of PC-AKI, concerns have been raised that contrast media is not an actual predisposing factor for PC-AKI. Previous studies have stated that there is no association between contrast media and renal function including the incidence of AKI, need for renal replacement therapy, and mortality in sepsis patients^27^ or intensive care unit patients^28,29^ and this has been seen in even a meta-analysis^30^. These conflicting results might be due to both the difficulties in defining or composing control groups with a retrospective study design and the near impossibility of performing a prospective randomized trial. However, the American College of Radiology clearly states in its manual on contrast media that PC-AKI is a rare, but real disease entity^31^. Hence, in addition to research on methods to prevent or attenuate PC-AKI, we also need to explore associations between contrast media and PC-AKI in the future.

There were a couple of limitations to this study. First, the expression of RAGE, TLR2 and CXCR4 which are known as receptors of HMGB1 on the cell surface, and NF-kB which is a downstream factor of HMGB1 were not evaluated in this study^14^. Second, the physiology and anatomy of rats and humans differ and these differences will need to be addressed before our findings can be applied in clinical practice. And lastly, only male rats were used in this experiment.

In conclusion, this study showed that HMGB1 plays an important role in the development of PC-AKI and that glycyrrhizin has a protective effect on renal function by inhibiting HMGB1 and reducing oxidative stress.

## Material and methods

### Animal preparation

Institutional Animal Care and Use Committee of Yonsei University Health System approved this experiment (ID number of 2019–0137) and all methods were carried out in accordance with relevant guidelines and regulations. Additionally, the animal experiment procedures were performed according to the ARRIVE guidelines. Twenty-four Sprague–Dawley male rats were used in this study (body weight:200-220 g). Rats were housed in an Animal Laboratory and maintained in a sterile 12 h light and 12 h dark experimental animal environment with 50 ± 100% humidity and 22 ± 2 °C temperature before the experiment^32^. Rats were classified into 3 groups: the control group (n = 8), PC-AKI group (n = 8) and PC-AKI with glycyrrhizin group (n = 8). There was no significant difference in body weight among the three groups (*P* = 0.190). At first, water was restricted for all groups for 16-24 h. In the PC-AKI model, 10 mg/kg of indomethacin (Sigma-Aldrich, St. Louis, MO) and 15 mg/kg of L-NAME (Cayman Chemical, Ann Arbor, MI) were administrated via an intraperitoneal injection (IP). After 20 min, 10 ml/kg of iodinated contrast media (Pamiray 370, Dongkook Lifescience, Seoul, South Korea) was administrated by intravenous injection (IV) through the tail vein^33^. In the PC-AKI with glycyrrhizin group, 30 mg/kg of glycyrrhizin (Selleckchem, Huston, TX) dissolved in saline was intraperitoneally administered 2 h before PC-AKI model construction. In all the other groups, the same volume of saline was administered but without glycyrrhizin. After 24 h, rats were anesthetized with 1.5% isoflurane in a mixture of nitrous oxide (0.7L/min) and oxygen (0.3L/min) and blood pressure was measured with the following methods: endotracheal intubation was done using a 16G angiocatheter (Becton, Dickinson and Company, Franklin lakes, NJ) and anesthesia was maintained with 1.5% isoflurane in a mixture of 80% nitrous oxide and 20% oxygen. The left inguinal area was shaved and rats were locally anesthetized using a local infiltrative anesthetic (0.2% Bupivacaine). Then, the left femoral artery was exposed and ligated distally with 4–0 black silk. After clipping the proximal part of the left femoral artery using a microclip, a small incision was made between the microclip and ligation with a microscissor. During microclip removal, an indwelling polyethylene-50 tube (Scientific Commodities Inc., Lake Havasu City, AZ) was inserted and pushed forward to the inguinal ligament level through the femoral artery. To measure arterial blood pressure, a left femoral arterial catheter was connected to the Lifewindow LW9x multi-parameter physiologic monitoring device (Digicare Biomedical Technology, Inc., Boston Beach, FL) through a 25 IU/ml heparin-filled invasive blood pressure transducer (Utah medical products, Inc., Midvale, Utha). The invasive blood pressure transducer was placed at heart level. After a stable and clear arterial waveform was obtained from the left femoral artery, the catheter was fixed with 4–0 black silk. Body temperature and end-tidal carbon dioxide were monitored using a rectal thermoprobe and capnography on a Lifewindow LW9x multi-parameter physiologic monitor to ensure the physiologic condition of the rat was constant during the entire procedure. Systolic pressure, diastolic pressure and calculated mean arterial pressure (MAP) were collected 3 min after finishing the procedure. There was no statistical difference among the 3 groups (*P* = 0.170) for blood pressure (Supplementary Figure 4). After blood pressure was measured, rats were euthanized using a CO_2_ incubator and the kidney tissue was harvested.

### Kidney function evaluation

After euthanasia, blood was collected without anticoagulants from a cardiac puncture of rats through the right atrium with a 26-gauge syringe needle and centrifuged at 3000 rpm for 20 min to obtain the serum. The serum levels of blood urea nitrogen (BUN), serum creatinine (SCr) and lactate dehydrogenase (LDH) were measured using a Cobas C502 autoanalyzer (Roche, Mannheim, Germany).

### Cell culture and contrast media exposure

The NRK52E cell line (immortalized normal rat renal proximal tubular cells) were cultured in DMEM with 5% FBS and 100U/ml penicillin–streptomycin at 37 °C in a humidified incubator with 5% CO_2_ for growth. When the cell confluence was 85%, the cells were exposed to 200 mg I/ml contrast media (Pamiray 370, Dongkook Lifescience, Seoul, South Korea) for 2 h. The glycyrrhizin pretreatment group was pretreated with 150 uM of glycyrrhizin (Selleckchem, Huston, TX) 90 min before contrast media exposure.

### RNA interference

We used siRNA that targeted HMGB1 to inhibit HMGB1 expression. The sense sequence was 5’-CUG CUU AGU UUA GGG AAC A-3’, and the antisense sequence was 5’-UGU UCC CUA AAC UAA GCA G-3’. HMGB1 siRNA was transiently transfected into NRK52E cells with Lipofectamin 2000 (Invitrogen, Carlsbad, CA).

### Quantitative real-time polymerase chain reaction

Total RNA was extracted from the kidney tissue using a commercial kit according to the manufacturer’s instructions (Hybrid-R kit 305–101, GeneAll Biotechnology, Seoul, South Korea)^34^. 1 μg of the total RNA was reverse transcribed with amfiRivert cDNA synthesis (GenDEPOT, Huston, TX) according to the manufacturer’s instructions. mRNA expression levels of HMGB1, KIM-1, NGAL, IL-1α, IL-1β, IL-6, IL-18 and caspase-3 were assessed using the SYBR-Green reagent (GenDEPOT, Huston, TX) with the ABI7500 real-time polymerase chain reaction (RT-PCR) system (Applied biosystem, Foster city, CA) and normalized to β-actin. Gene expression was quantified using β-actin as the internal loading control. All RT-PCR reactions were duplicated and threshold (Ct) values were analyzed by the 2^(−ΔΔCt)^ method. The primer sequences for real-time PCR are given in Table 1.

**Table 1:** Primer sequences.

Target gene	Forward sequence (5′–3′)	Reverse sequence (5′–3′)
β-actin	TGGCACCCAGCACAATGAA	CTAAGTCATAGTCCGCCTAGAAGCA
KIM-1	AACGCAGCGATTGTGCATCC	GTACACTCACCATGGTAACC
NGAL	GATGAACTGAAGGAGCGATTC	TCGGTGGGAACAGAGAAAAC
IL-18	AAACCCGCCTGTGTTCGA	ATCAGTCTGGTCTGGGATTCGT
HMGB1	ATGGGCAAAGGAGATCCTA	ATTCTCATCATCTCTTCT
IL-1α	CTCTAGAGCACCATGCTACAGAC	TGGAATCCAGGGGAAACACTG
IL-1β	ATGGCAACTGTTCCTGAACTCAACT	CAGGACAGGTATAGATTCTTTCCTTT
IL-6	AGGATACCACTCCCAACAGACCT	CAAGTGCATCATCGTTGTTCATAC
TNF-α	AGCCCTGGTATGAGCCCATGTA	CCGGACTCCGTGATGTCTAAG
Caspase-3	ACTGGAAAGCCGAAACTCTTCATCA	GGAAGTCGGCCTCCACTGGTATC

### Western blotting

The harvested kidney tissue was homogenized and lysed in a pro-prep extraction solution (iNtRON Biotechnologist, Seong-nam, Korea) followed by protein quantitation with the Bradford method^34^. In addition, nucleus and cytoplasmic proteins were extracted using NE-PER Nuclear and Cytoplasmic Extraction reagents (Thermo Scientific, Rockford, IL, USA). Lysates were fractioned on 10–15% sodium dodecyl sulfate (SDS)-polyacrylamide gels and transferred to polyvinylidene difluoride membranes. The membranes were incubated overnight at 4 °C with primary antibodies against HMGB1 (1:1000, #ab18256, Abcam, Cambridge, MA), cleaved caspase-3 (1:1000, #9664, Cell signaling, MA, USA) β-actin (1:10000, #LF-PA0207, AbFrontier, Seoul, South Korea) and LaminB1 (1:1000, #12586, Cell signaling, MA, USA). After the membranes were washed three times in 1xTBS-T for 15 min each, they were incubated with HRP-conjugated secondary antibodies (goat anti-rabbit IgG-HRP, 1:10000 #SA002-500, GenDepot, Houston, TX) for 1 h at room temperature. Then the membranes were washed three times in 1xTBS-T for 15 min again. The blotted membranes were visualized by ECL reagents and exposed to X-ray film. The results were normalized to the β-actin and LaminB1 loading control and band density was measured using Image J software (National Institutes of Health, Bethesda, MD, https://imagej.nih.gov/ij)^34^.

### Enzyme-linked immunosorbent assay (ELISA)

To measure rat serum HMGB1, IL-18 and urinary KIM-1, we used the Rat HMGB1 ELISA kit (E-EL-R0505, Elabscience, Houston, Tx), Rat IL-18 ELISA kit (ab213909) and Rat KIM-1 ELISA kit (ab223858, Abcam, Cambridge, MA) according to the manufacturer’s instructions. Absorbance levels of the concentrations were measured at 450 nm using a microplate reader (Molecular Devices, Sunnyvale, CA).

### Determination of MDA concentration

Membrane lipid peroxidation was assessed by measuring malondialdehyde (MDA), a product of membrane lipid peroxidation, using 2-thiobarbituric acid reactive substances. The MDA levels were calculated as MDA equivalents using a commercial kit Cat#10009055 (Cayman Chemical, Ann Arbor, MI) according to the manufacturer’s instructions^34^.

### Histology, immunofluorescence and H_2_DCFDA staining

Kidney tissue was fixed with 10% buffered formalin embedded in paraffin, and cut into 4 μm sections. The paraffin-embedded tissues were deparaffinized by xylene and rehydrated in increasing concentrations of ethanol (70%, 90%, 95%, 100%). Then, H&E staining was performed using the Leica Autostainer. Renal tubular damage was assessed with the following score system: score of 0 (no injury); score of 1 (< 10%, minimal); score of 2 (10–25%, mild); score of 3 (25–50%, moderate); score of 4 (50–74%, severe), and score of 5 (> 75%, very severe)^35^. For H_2_DCFDA staining, the sectioned slide was deparaffinized and incubated with 10 μM H_2_DCFDA for 15 min in a 37 °C incubator. The stained slides were observed with a confocal microscope (LSM700, Carl Zeiss GmbH, Jena, Germany).

### TUNEL staining

To determine the degree of apoptosis in the kidney, we performed the terminal deoxynucleotidyl transferase (TdT)-mediated dUTP nick-end-labeling (TUNEL) assay using the TACS 2 TdT-DAB in situ apoptosis detection kit Cat#4810–30-K (Trevigen inc., Gaithersburg, MD, USA) according to the manufacturer’s instructions.

### Transmission electron microscopy (TEM)

Kidney tissue was fixed overnight in 0.1 M phosphate buffer (PH7.4) containing 2% glutaraldehyde, 2% paraformaldehyde and 0.5% CaCl_2_. The samples were washed twice with 0.1 M phosphate buffer for 30 min and were then fixed for 2 h with 1% OsO_4_ dissolved in 0.1 M phosphate buffer, followed by dehydration through a series of increasing ethanol concentrations (50–100% with increments of 10%) with 10 min being allocated for each concentration. Specimens were embedded by poly/Bed812 kit (Polyscience, Inc., Warrington, PA) and polymerized at 65 °C in an electron micro-oven (TD-700, DOSAKA, Kyoto, Japan) for 24 h^32^. Blocks were cut using an ultramicrotome (LEICA EM UC-7, Leica Microsystem, Vienna, Austria) and observed with TEM (JEM-1011, JEOL, Tokyo, Japan). To assess the effect the contrast media and glycyrrhizin had on cells, we evaluated cellular vacuolization by performing TEM with the same image magnification (5 K) and the number of vacuoles within the tubular cell in the field of view of each TEM image were counted by an experienced researcher who was blinded to the study results.

### Statistical analysis

All results were presented as means ± standard errors of the mean (SEMs). Statistical analyses were performed using Prism 8.3.0 (Graphpad, San Diego, CA). One-way analysis of variance (ANOVA) and post-hoc comparisons (Bonferroni test) were performed to compare the 3 groups. *P* value of less than 0.05 was considered to indicate a statistically significant difference. All *P*-values were two-sided.

## Supplementary Information

Supplementary Figures.
