# Supplementary material for: Protective effect of glycyrrhizin, a direct HMGB1 inhibitor, on post-contrast acute kidney injury
Source: Sci Rep. 2021 Aug 2;11:15625. doi: 10.1038/s41598-021-94928-5 (PMC8329191; doi:10.1038/s41598-021-94928-5)
Supplement: Supplementary file 1 — Supplementary Figures. [file 41598_2021_94928_MOESM1_ESM.pdf]

Supplementary information for:

Protective effect of glycyrrhizin, a direct HMGB1 inhibitor, on post-contrast acute kidney injury

Hyewon Oh, MS[1], Arom Choi, MD, MS[2], Nieun Seo, MD, PhD[1], Joon Seok Lim, MD, PhD,[1] Je Sung You, MD, PhD[2*], Yong Eun Chung, MD, PhD[1*]

[1]Department of Radiology, Yonsei University College of Medicine, Seoul, Republic of Korea

[2]Department of Emergency Medicine, Yonsei University College of Medicine, Seoul, Republic of Korea

* Je Sung You and Yong Eun Chung contributed equally to this work;

Correspondence:

Yong Eun Chung, MD, PhD

Department of Radiology, Severance Hospital, Yonsei University College of Medicine, 50-1 Yonsei-ro, Seodaemun-gu, Seoul 03722, Republic of Korea, Tel: 82-2-2228-7400, Fax: 82-2-2227-8337, E-mail: yelv@yuhs.ac

Je Sung You, MD, PhD

Department of Emergency Medicine, Yonsei University College of Medicine, 211 Eonju-Ro, Gangnam-Gu, Seoul 06273, Republic of Korea, Tel: 82-2-2019-3030, Fax: 82-2-2019-4820, E-mail: youjsmd@yuhs.ac

Supplementary Figure 1. Full- length blots of Figure 2A and Figure 5B

__Figure 2A__

A. Total protein HMGB1

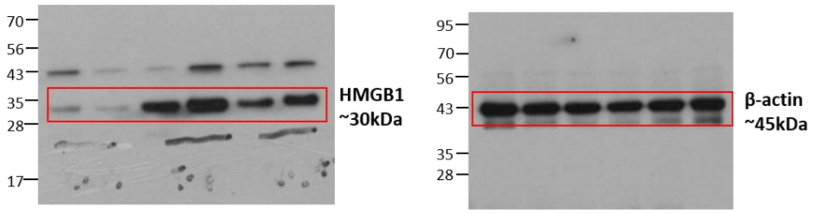

B. Nucleus HMGB1

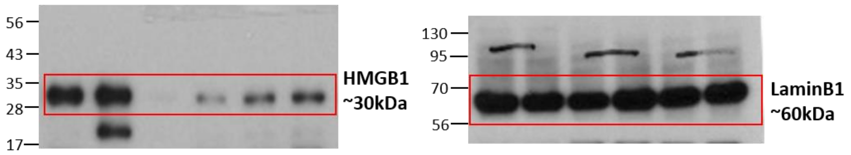

C. Cytoplasmic HMGB1

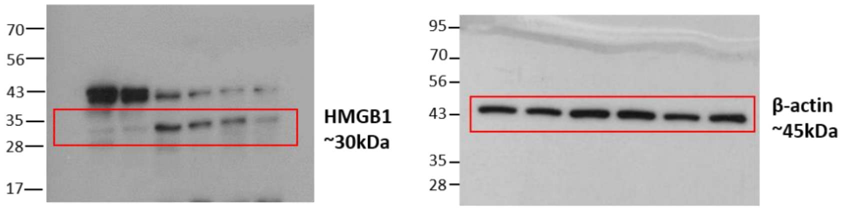

__Figure 5B__

D. Cleaved caspase-3

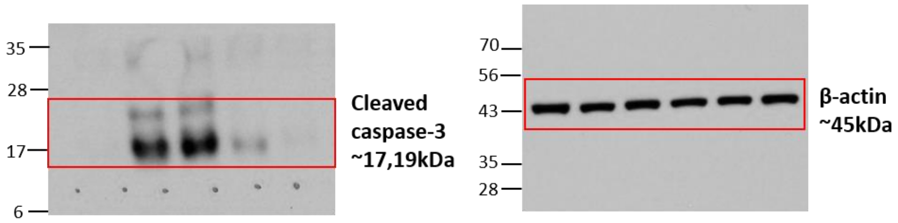

Supplementary Figure 1. Full-length blots of Figure 2A and Figure 5B. The blots were loaded with rat kidney tissue and incubated with antibodies against HMGB1, β-actin and LaminB1 to show total protein (A), nucleus (B), cytoplasmic (C) HMGB1. (D) Full-length blots of cleaved caspase-3 and β-actin in Figure 5B. The membrane exposure time was different for each antibody. Dashed lines represent the cropped region shown in Figure 2A and 5B.

Supplementary Figure 2

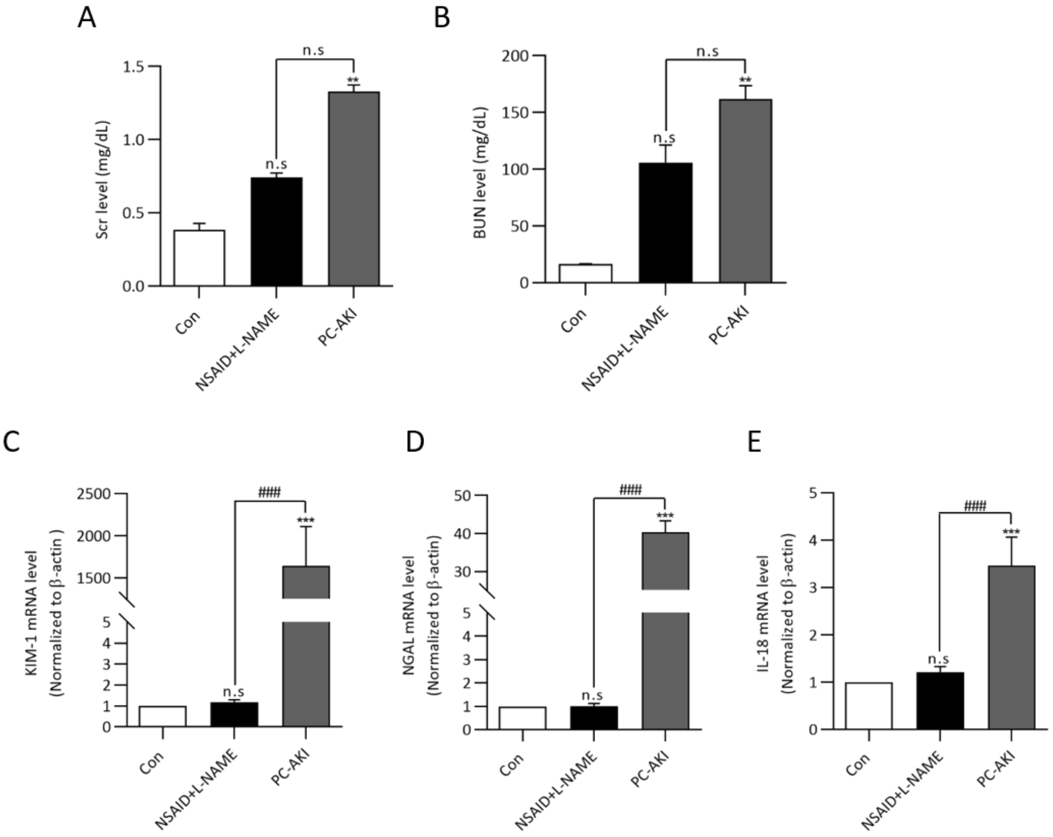

Supplementary Figure 2. (A-B) Compared to the controls, Scr and BUN significantly increased in the PC-AKI groups, whereas there was no difference between the controls and the NSAID+L-NAME groups. (C-E) Relative expressions of KIM-1, NGAL and IL-18 mRNA were analyzed using RT-PCR. All kidney injury markers were significantly higher in PC-AKI compared to both the controls and the NSAID+L-NAME group, whereas there was no difference between the controls and the NSAID + L-NAME group. Results were expressed as means±SEMs. N=4 for each group. Statistical significance: $^{**}P<0.01$ and $^{***}P<0.001$ Con vs PC-AKI, $^{###}P<0.001$ NSAID+L-NAME vs PC-AKI. Abbreviation: PC-AKI; post-contrast acute kidney injury

Supplementary Figure 3

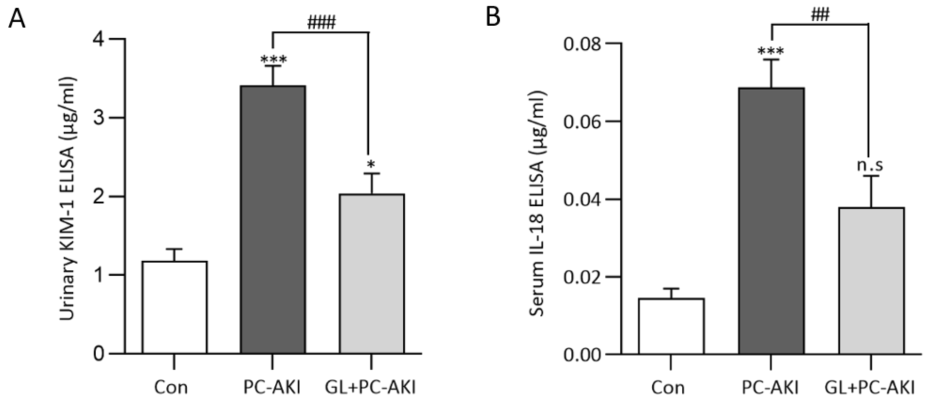

Supplementary Figure 3. Effect on glycyrrhizin on kidney injury marker in urinary and serum using ELISA. KIM-1 in urine (A) and serum IL-18 (B) was significantly higher in PC-AKI group compared to control group, where they were significantly lower in PC-AKI with glycyrrhizin group compared to PC-AKI group. Statistical significance: $^{*}P<0.05$ and $^{***}P<0.001$ Con vs PC-AKI and Con vs PC-AKI+GL, $^{\#\#}P<0.01$ and $^{\#\#\#}P<0.001$ PC-AKI vs PC-AKI+GL. Abbreviation: PC-AKI; Post-contrast acute kidney injury, GL; glycyrrhizin, ELISA; Enzyme-Linked immunosorbent Assay.

Supplementary Figure 4. Blood pressure

	SBP	DBP	MAP
Con	133.5±8.5	119.3±6.9	124.0±7.5
PC-AKI	123.3±7.0	104.8±14.1	110.9±11.5
GL+PC-AKI	127.0±7.3	104.5±11.9	112.0±20.9

Abbreviation; Post contrast-acute kidney injury; PC-AKI, Glycyrrhizin; GL, Systolic blood pressure; SBP, Diastolic blood pressure; DBP, Mean arterial pressure; MAP. MAP measurement calculation; DBP+1/3(SBP-DBP). No statistical difference among 3 groups.
